# Laryngeal Synovial Sarcoma—A Rare Entity With an Uncommon Clinical Manifestation of Hemoptysis: A Case Report and Review of the Literature

**DOI:** 10.1155/crot/4214277

**Published:** 2026-07-20

**Authors:** Kamyar Iravani, Negar Azarpira, Mohammadreza Akhtari, Sajjad Soltani, Nazila Shirvani, Pooria Zare

**Affiliations:** ^1^ Department of Otolaryngology, Otolaryngology Research Center, Shiraz University of Medical Sciences, Shiraz, Iran, sums.ac.ir; ^2^ Transplant Research Center, Shiraz University of Medical Sciences, Shiraz, Iran, sums.ac.ir; ^3^ Department of Pathology, School of Medicine, Shiraz University of Medical Sciences, Shiraz, Iran, sums.ac.ir

**Keywords:** cancer, chemoradiotherapy, chemotherapy, dysphonia, larynx, oncology, radiotherapy, synovial sarcoma

## Abstract

**Background:**

Synovial sarcoma is a mesenchymal malignancy that most commonly arises in the lower extremities. Fewer than 5% of cases originate in the head and neck, and laryngeal involvement is exceptionally rare.

**Case Presentation:**

A 22‐year‐old man presented with a 6‐month history of progressive hoarseness and hemoptysis. Direct laryngoscopy was performed, and biopsies were obtained. Histopathological examination established the diagnosis of laryngeal synovial sarcoma. The patient received three cycles of neoadjuvant chemotherapy, followed by total laryngectomy with bilateral neck dissection. Adjuvant radiotherapy was initiated postoperatively, and the patient remained recurrence‐free at 12 months of follow‐up.

**Discussion:**

Fewer than 5% of synovial sarcomas occur in the head and neck, and laryngeal involvement is rare. Three histopathological subtypes are recognized: monophasic, biphasic, and poorly differentiated. Immunohistochemistry and detection of the t(X; 18) translocation play a key role in diagnosis. Surgical resection remains the mainstay of treatment, although chemoradiotherapy may also be beneficial. Recurrence is a major concern, with local recurrence reported in up to 45% of patients and distant metastasis in approximately 33%.

**Conclusion:**

Because laryngeal synovial sarcoma is rare, reporting such cases may improve future diagnosis, evaluation, and management of this uncommon entity.

## 1. Introduction

Synovial sarcoma is a rare and aggressive soft tissue tumor of mesenchymal cell origin that usually arises from the upper and lower extremities. However, fewer than 5% of synovial sarcomas have a head and neck origin, and laryngeal involvement is rare [1, 2]. The tumor most commonly occurs in the third decade of life and shows a male predominance [3]. This study aims to present a case of laryngeal synovial sarcoma in a patient who was referred to our ENT referral center in February 2025.

## 2. Case Presentation

A 22‐year‐old man presented with a chief complaint of hemoptysis of 6 months’ duration. He also reported progressive hoarseness, dyspnea, dysphagia, and recent weight loss. His past medical and surgical history was unremarkable. However, he had a positive family history: his uncle had died at the age of 70 from laryngeal synovial sarcoma, having undergone chemoradiotherapy without surgical resection. There was no history of smoking or alcohol consumption. On physical examination of the head and neck, no lymphadenopathy or neck swelling was detected.

Direct laryngoscopy revealed an erythematous and hemorrhagic mass within the right pyriform sinus, whereas the vallecula, epiglottis, arytenoid, and aryepiglottic fold appeared normal (Figure 1).

**FIGURE 1:**
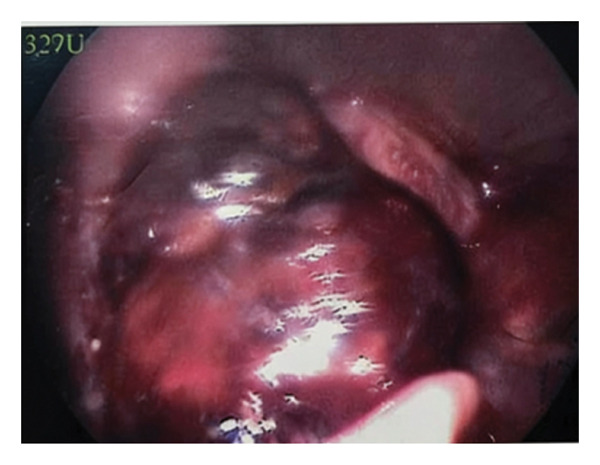
Direct laryngoscopy view shows an erythematous and hemorrhagic mass lesion in the right pyriform fossa.

Biopsies were taken from the mass and sent to the pathology laboratory, where a malignant biphasic neoplasm consistent with synovial sarcoma was diagnosed.

An immunohistochemical study was performed, and the markers AE1/AE3, EMA, MIC2, calretinin, and TLE1 were positive, whereas 34βE12 was negative. In addition, a FISH study demonstrated the SS18–SSX fusion mutation, which is highly specific for synovial sarcoma.

Contrast‐enhanced neck CT showed an infiltrating soft tissue lesion with heterogeneous enhancement in the right supraglottic region, measuring approximately 25 × 15 mm and suggestive of a malignant tumor, together with several nonspecific lymph nodes in different neck compartments, the largest measuring up to 10.5 × 8 mm in the right upper jugular chain (Figure 2). Contrast‐enhanced CT of the chest and abdominopelvic region was unremarkable.

**FIGURE 2:**
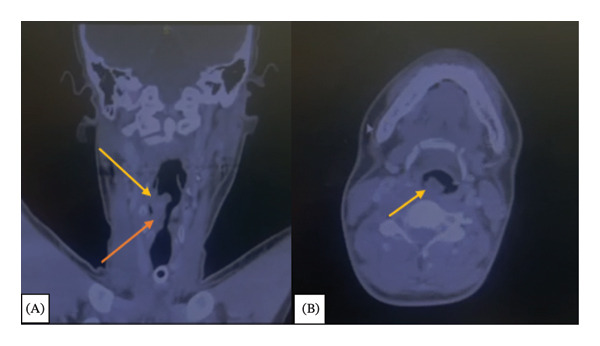
(A) Coronal and (B) axial views of the neck CT scan show a soft tissue lesion measuring about 25 × 15 mm in the right supraglottic region.

The apparent discrepancy between the endoscopic and radiological findings reflects the anatomical proximity of these subsites: the tumor was centered in the right supraglottic region and extended inferolaterally into the medial wall of the right pyriform sinus. Consequently, the most prominent component was visualized within the pyriform sinus on direct laryngoscopy, whereas contrast‐enhanced CT demonstrated the bulk of the lesion in the supraglottic region.

The patient had initially received three cycles of chemotherapy before surgery. After referral to our center, a bilateral neck dissection was performed, and all resected nodal specimens were submitted for histopathological examination. On preoperative contrast‐enhanced CT, suspicious nodes had been identified within the right upper jugular chain (Level III), the principal site of regional lymphatic drainage for supraglottic laryngeal tumors. All submitted nodes, including the right‐sided Level III (Zone 3) group, were free of metastatic disease. A right thyroid lobectomy was also performed. Subsequently, the patient underwent a total laryngectomy, during which the larynx was removed with free margins (Figure 3). Pharyngoplasty and tracheostomy were also performed.

**FIGURE 3:**
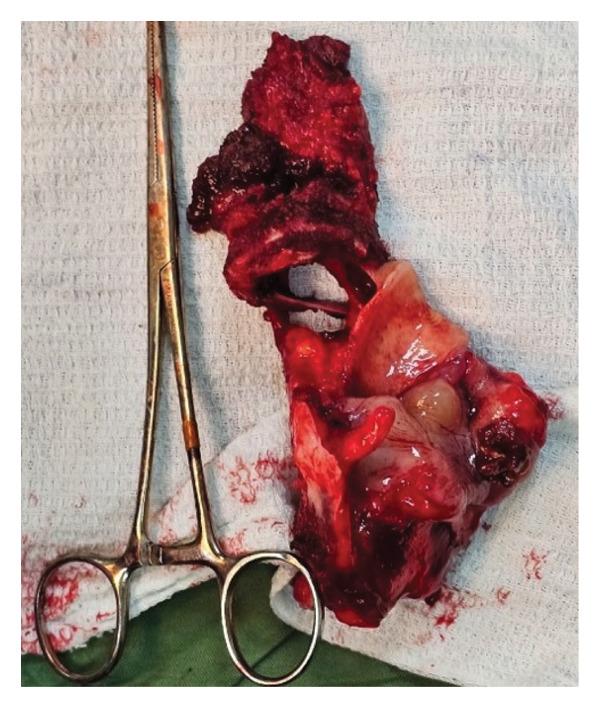
Photograph of resected larynx with a free margin and brownish mass lesion in the right pyriform fossa (1 × 1 × .8 cm).

The specimens were sent for pathological evaluation. Grossly, a brown mass lesion measuring approximately 1 × 1 × 0.8 cm was observed in the right pyriform sinus (Figure 4), and histopathological evaluation confirmed a biphasic synovial sarcoma (spindle and epithelioid types) without necrosis or lymphovascular invasion (Figures 5 and 6). The proximal and distal resection margins were free of tumor. There was no evidence of nodal involvement or metastasis. The mitotic rate was approximately 0–1 per 10 high‐power fields. Because the patient had received neoadjuvant chemotherapy before resection, the tumor was staged according to the 8th edition of the AJCC staging system as ypT1 N0 M0 (histologic grade G2, FNCLCC).

**FIGURE 4:**
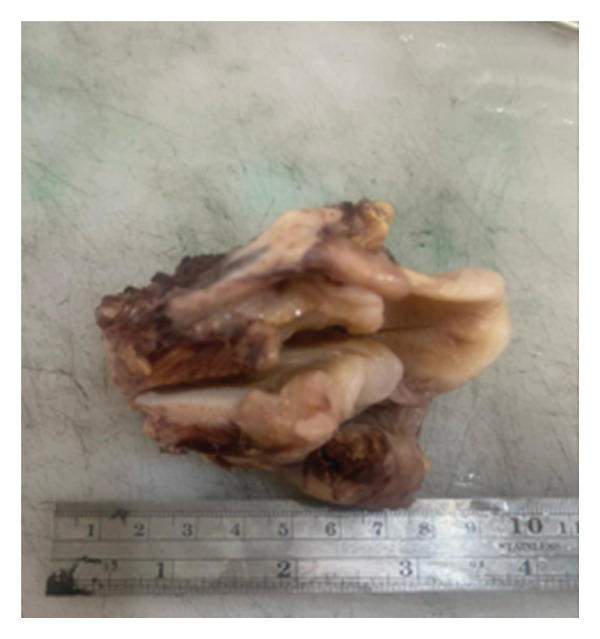
Gross pathologic appearance of the resected larynx measuring about 8 × 5 × 4 cm, with the brown discolored area at the right piriform sinus (1 × 1 × .8 cm).

**FIGURE 5:**
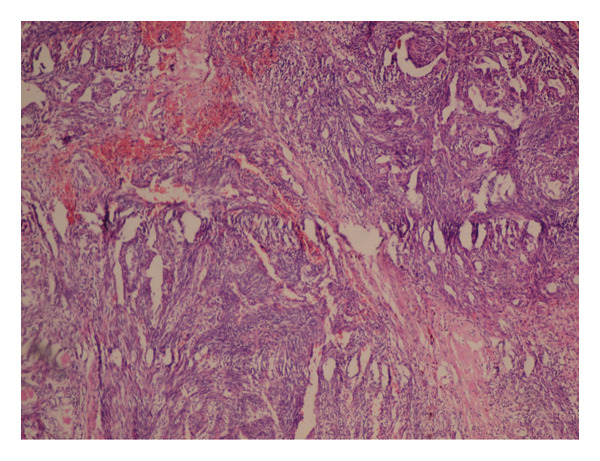
Low‐power histologic view of an infiltrative biphasic malignant tumor composed of spindle cells and gland‐like epithelial structures (H&E, 40 × magnification).

**FIGURE 6:**
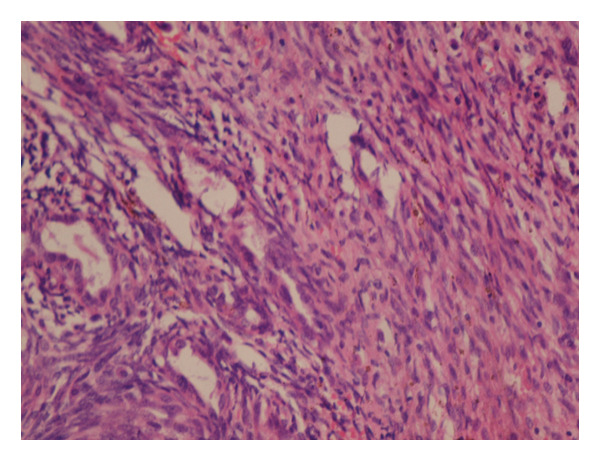
High‐power histologic view of hypercellular spindle cells arranged in a fascicular pattern, adjacent to a gland‐like structure with epithelioid morphology (H&E, 200 × magnification).

The patient was discharged with good swallowing function and no signs of aspiration. Postoperative radiotherapy was initiated. He has since remained under routine oncologic follow‐up for the past 12 months, with no evidence of recurrence.

## 3. Discussion and Review of Articles

Synovial sarcoma originates from pluripotent mesenchymal stem cells and most frequently occurs in the lower limb. The head and neck are uncommon sites; within this region, it may arise in the hypopharynx, oropharynx, trachea, paranasal sinuses, and, rarely, the larynx [4, 5].

Patients usually present with hoarseness and, less frequently, dyspnea. Other features, such as dysphagia or neck swelling, may also occur [6].

Histopathologically, three morphological subtypes are recognized: a monophasic form, composed only of spindle cells; a biphasic form, containing both glandular and epithelial elements; and a poorly differentiated form, consisting of uniform, densely packed small cells [4, 7].

The reciprocal translocation t(X; 18), which results in fusion of the SYT (SS18) gene on chromosome 18 with the SSX1, SSX2, or SSX4 gene on chromosome *X*, is highly specific for the diagnosis of synovial sarcoma [7, 8].

On immunohistochemistry, epithelial markers such as cytokeratin 7 and 19, epithelial membrane antigen (EMA), vimentin, Bcl‐2, and TLE1 may be positive, whereas CD34 and FLI‐1 are typically negative [7–9].

Radiological imaging also plays an important diagnostic role. On CT, synovial sarcoma may appear as a hypodense lesion with areas of calcification. On MRI, the lesion is typically isointense to skeletal muscle on T1‐weighted images, whereas on T2‐weighted images it may be hyperintense or display the “triple sign,” showing hyperintense, isointense, and hypointense areas simultaneously [9, 10].

The management of synovial sarcoma is multimodal, and some controversy remains regarding the optimal approach. Radical surgical resection with adequate margins is considered the mainstay of treatment [7, 8, 10].

Adjuvant chemotherapy, usually with ifosfamide and doxorubicin, may be beneficial in cases with involved margins, high‐grade tumors, or distant metastasis, and may improve disease‐specific survival (DSS) [11, 12].

Adjuvant radiotherapy may be useful in cases with high‐grade lesions, positive surgical margins, tumors larger than 5 cm, or tumor recurrence [10].

In addition, some studies suggest that neoadjuvant chemotherapy may be beneficial for tumors larger than 5 cm or those located in high‐risk sites [12].

Prognosis depends on multiple factors, including patient age, tumor size and extension, neurovascular invasion, high mitotic rate, and the extent of necrosis [10, 13]. Disease recurrence is a major concern: approximately 45% of patients may develop local recurrence, and 33% may present with metastatic disease [13].

In a systematic review by Shein et al., which evaluated 39 cases of laryngeal synovial sarcoma reported between 1975 and 2017, the mean patient age was approximately 32 years, with a male predominance (69.2%). The most common tumor location was the supraglottic structures, including the aryepiglottic folds, arytenoids, and epiglottis. On histopathology, most cases showed biphasic morphology (80.7%). FISH analysis was performed in 8 cases and was positive in all of them [7]. Surgical resection, the principal treatment, was performed in all patients (10 total laryngectomies and 10 partial laryngectomies). Radiotherapy was administered in more than half of the patients (*n* = 21), most commonly as adjuvant therapy. Over a mean follow‐up of approximately 3.2 years, disease recurrence was observed in 12.8% (*n* = 5) of cases [7].

As summarized in Table 1, we have collated the epidemiological and histopathological findings of case reports of laryngeal synovial sarcoma published between 2018 and 2025. The mean patient age was approximately 21 years (range: 9–31). Consistent with the male predominance of the disease, 6 cases were men and only 2 were women. The supraglottic area was the most common site of involvement, including the aryepiglottic fold, pyriform sinus, and arytenoid. On histopathology, most cases showed a monophasic spindle‐cell form (*n* = 5); 2 cases were of the biphasic subtype, and 1 case was classified as poorly differentiated. The t(X; 18) translocation was assessed in 3 cases and was positive in all of them. All eight patients underwent surgical resection: total laryngectomy in 5, partial laryngectomy in 1, and CO_2_ laser‐assisted partial pharyngectomy in 1. Most patients received adjuvant radiotherapy (*n* = 7), while chemotherapy was administered more commonly as adjuvant (*n* = 3) than as neoadjuvant therapy (*n* = 1) [8, 10, 12, 14, 15]. The mean follow‐up period was approximately 4.5 years. During follow‐up, 7 patients were alive and recurrence‐free; however, one patient developed local recurrence in the nasopharyngeal area and was subsequently lost to follow‐up [16].

**TABLE 1:** Comparison of different epidemiologic and histologic findings of laryngeal synovial sarcoma case reports from 2018 to 2025.

First author/year	Country	Age	Sex	Site of the tumor	Histologic subtype	Translocation t(x,18)	Management	Follow up
Madabhavi/2018 [14]	India	31	Male	Left arytenoid, left pyriform, left aryepiglottic fold	Monophasic	—	Surgical resection, adjuvant chemoradiotherapy	Recurrence‐free after 3 months
Bellakhdhar/2018 [8]	Tunisia	27	Male	Right aryepiglottic and right pyriform sinus	Poorly differentiated	—	Total laryngectomy/adjuvant radiotherapy	Recurrence‐free after 10 years
Bellakhdhar/2018 [8]	Tunesia	18	Male	Left aryepiglottic fold	Monophasic	—	Total laryngectomy/adjuvant radiotherapy	Recurrence‐free after 2 years
Kumar/2019 [12]	India	28	Female	Right vocal cord and right aryepiglottic fold	Monophasic	—	CO_2_ laser‐assisted partial pharyngectomy, adjuvant chemoradiotherapy	Recurrence‐free after 18 months
Shein/2021 [15]	Australia	9	Male	Left lateral aspect of thyroid cartilage	Biphasic	Present	Partial laryngectomy/neck dissection/adjuvant radiotherapy	Recurrence‐free after 6 years
Alhatem/2019 [16]	USA	10	Male	Left supraglottic, aryepiglottic fold	Monophasic	Present	Total laryngectomy/adjuvant chemoradiotherapy	Local recurrence in the nasopharynx after 15 years, then lost follow‐up
Perez/2024 [10]	Mexico	26	Female	Glottis/subglottic area	Monophasic	—	Total laryngectomy	Recurrence‐free after 6 months
Current study	Iran	22	Male	Right pyriform fossa	Biphasic	Present	Total laryngectomy/neck dissection/neoadjuvant chemotherapy/adjuvant radiotherapy	Recurrence‐free after 3 months

## 4. Conclusion

Because synovial sarcoma rarely occurs in the larynx, identifying and reporting such cases may improve our ability to diagnose, evaluate, and manage this condition in the future. Early diagnosis and a multidisciplinary treatment approach can improve patient survival, and close follow‐up plays an important role in detecting recurrence and metastasis.

## Funding

No funding was received for this manuscript.

## Ethics Statement

Ethical approval for this case report was obtained from the Ethics Committee of Shiraz University of Medical Sciences. This case report complies with the ethical standards required for medical case reports.

## Consent

Written informed consent was obtained from the patient for the publication of this case report and the accompanying images. The patient’s identity has been anonymized to maintain confidentiality.

## Conflicts of Interest

The authors declare no conflicts of interest.

## Data Availability

The data supporting the findings of this study are available from the corresponding author on request. The data are not publicly available owing to privacy and ethical restrictions.
